# Bis[chloridobis(1,10-phenanthroline)copper(II)] penta­cyanido­nitro­soferrate(II) di­methyl­formamide monosolvate

**DOI:** 10.1107/S1600536813015547

**Published:** 2013-06-15

**Authors:** Julia A. Rusanova, Olesia V. Kozachuk, Viktoriya V. Dyakonenko

**Affiliations:** a Taras Shevchenko National University, Department of Chemistry, Volodymyrska str. 64/13, 01601 Kyiv, Ukraine; b STC, "Institute for Single Crystals", National Academy of Sciences of Ukraine, Lenina ave. 60, Kharkov 61001, Ukraine

## Abstract

The title complex [CuCl(C_12_H_8_N_2_)_2_]_2_[Fe(CN)_5_(NO)]·C_3_H_7_NO, consists of discrete [Cu(phen)_2_Cl]^+^ cations (phen is 1,10-phenanthroline), [Fe(CN)_5_NO]^2−^ anions and one di­methyl­formamide (DMF) solvent mol­ecule of crystallization per asymmetric unit. The Cu^II^ atom is coordinated by two phenanthroline ligands and one chloride ion in a distorted trigonal–bipyramidal geometry. The dihedral angle between the phen ligands is 77.92 (7)°. The cation charge is balanced by a disordered nitro­prusside counter-anion with the Fe^II^ atom located on an inversion center with a slightly distorted octa­hedral coordination geometry. In the crystal, weak C—H⋯N and C—H⋯Cl hydrogen bonds connect anions and cations into a two-dimensional network parallel to (100). In addition, π–π stacking inter­actions are observed with centroid–centroid distances in the range 3.565 (2)–3.760 (3)Å. The di­methyl­formamide solvent mol­ecule was refined as disordered about an inversion center.

## Related literature

For background to the direct synthesis of coordination compounds, see: Buvaylo *et al.* (2005 ▶); Makhankova *et al.* (2002 ▶); Nesterova *et al.* (2004 ▶, 2005 ▶, 2008 ▶); Pryma *et al.* (2003 ▶); Vinogradova *et al.* (2002 ▶); Vassilyeva *et al.* (1997 ▶). For the structures of related complexes, see: Nikitina *et al.* (2008 ▶); Vreshch *et al.* (2009 ▶); Onawumi *et al.* (2010 ▶); Sui *et al.* (2006 ▶); Xiao *et al.* (2004 ▶); Soria *et al.* (2002 ▶); Shevyakova *et al.* (2002 ▶).
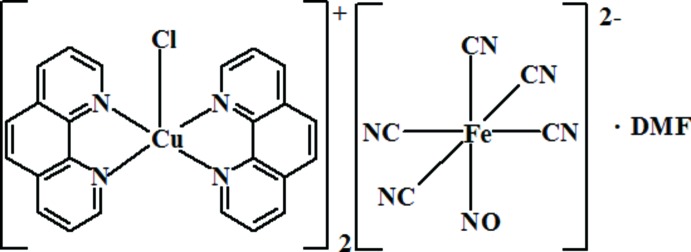


## Experimental

### 

#### Crystal data

[CuCl(C_12_H_8_N_2_)_2_]_2_[Fe(CN)_5_(NO)]·C_3_H_7_NO
*M* *_r_* = 1207.85
Triclinic, 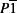

*a* = 9.9645 (13) Å
*b* = 10.6001 (18) Å
*c* = 12.623 (2) Å
α = 79.585 (14)°
β = 84.896 (12)°
γ = 82.047 (12)°
*V* = 1295.9 (4) Å^3^
*Z* = 1
Mo *K*α radiation
μ = 1.25 mm^−1^
*T* = 293 K
0.50 × 0.40 × 0.20 mm

#### Data collection

Oxford Diffraction Xcalibur 3 diffractometer
Absorption correction: numerical (*CrysAlis PRO*; Oxford Diffraction, 2010 ▶) *T*_min_ = 0.575, *T*_max_ = 0.789
11289 measured reflections
5647 independent reflections
2685 reflections with *I* > 2σ(*I*)
*R*_int_ = 0.041

#### Refinement

*R*[*F* ^2^ > 2σ(*F* ^2^)] = 0.061
*wR*(*F* ^2^) = 0.182
*S* = 0.99
5647 reflections
366 parameters
5 restraints
H-atom parameters constrained
Δρ_max_ = 0.73 e Å^−3^
Δρ_min_ = −0.61 e Å^−3^

### 

Data collection: *CrysAlis PRO* (Oxford Diffraction, 2010 ▶); cell refinement: *CrysAlis PRO*; data reduction: *CrysAlis PRO*; program(s) used to solve structure: *SHELXTL* (Sheldrick, 2008 ▶); program(s) used to refine structure: *SHELXTL*; molecular graphics: *SHELXTL* and *PLATON* (Spek, 2009 ▶); software used to prepare material for publication: *publCIF* (Westrip, 2010 ▶).

## Supplementary Material

Crystal structure: contains datablock(s) I, New_Global_Publ_Block. DOI: 10.1107/S1600536813015547/lh5620sup1.cif

Structure factors: contains datablock(s) I. DOI: 10.1107/S1600536813015547/lh5620Isup2.hkl

Additional supplementary materials:  crystallographic information; 3D view; checkCIF report

## Figures and Tables

**Table 1:** Hydrogen-bond geometry (Å, °)

*D*—H⋯*A*	*D*—H	H⋯*A*	*D*⋯*A*	*D*—H⋯*A*
C12—H12⋯Cl1^i^	0.93	2.82	3.701 (6)	159
C23—H23⋯N6^ii^	0.93	2.52	3.425 (7)	163

Symmetry codes: (i) 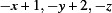; (ii) 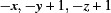.

**Fig. 1.**
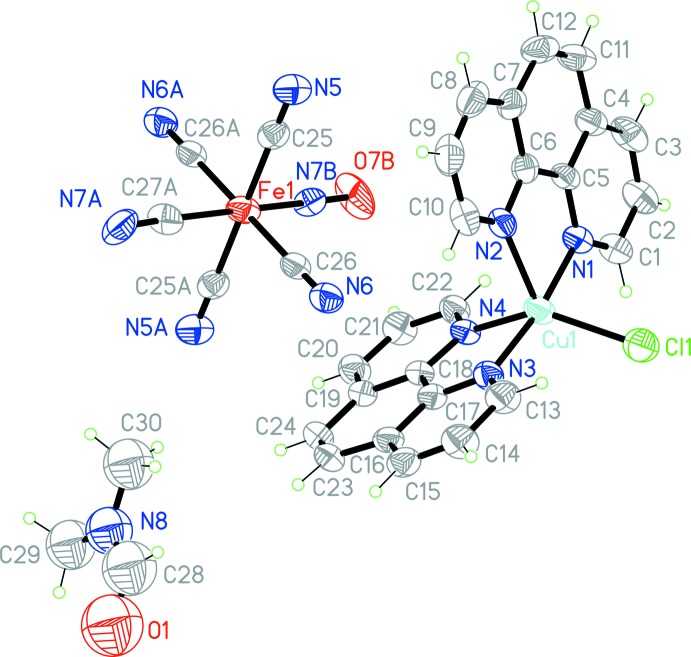
The molecular structure of the title compound with displacement ellipsoids drawn at the 50% probability level. Symmetry code (A): -x+1, -y+1, -z+1.

**Fig. 2.**
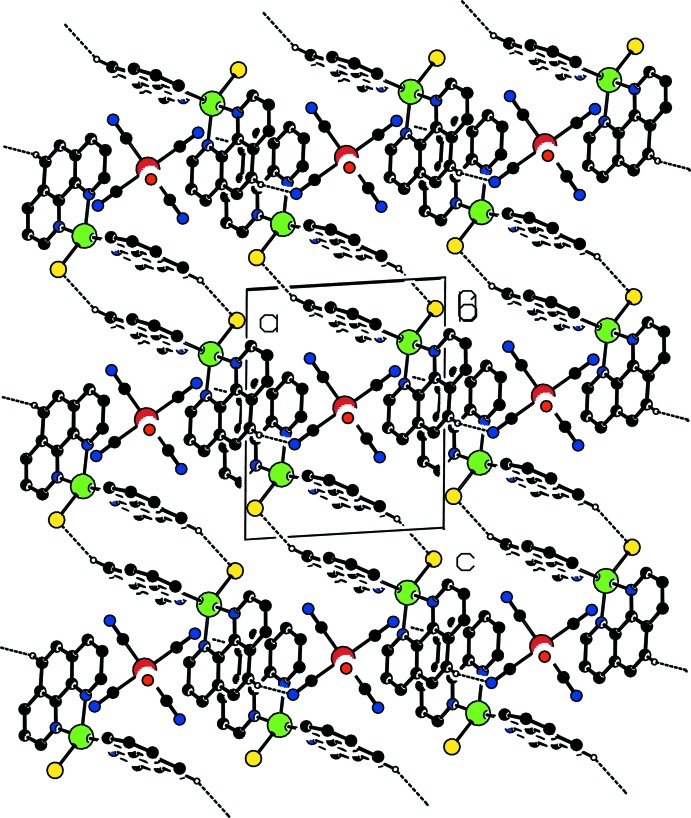
Part of the crystal structure with weak hydrogen bonds shown as dashed lines. Only H atoms involved in hydrogen bonds are shown. The disordered solvent molecule is not shown as it is not involved in the hydrogen bond motif.

## supplementary crystallographic information

### Comment

This work is a continuation of our research in the field of direct synthesis of coordination compounds (Buvaylo *et al.*, 2005; Makhankova *et al.*, 2002; Nesterova *et al.*, 2004,2005,2008; Pryma *et al.*, 2003; Vinogradova *et al.*, 2002; Vassilyeva *et al.*, 1997). It was shown recently the possibility of using anionic complexes as a source of metalloligands or the second metal in direct synthesis of heterometallic compounds (Nikitina *et al.*, 2008; Vreshch *et al.*, 2009).

In this paper we present a novel Cu/Fe heterometallic ionic complex [CuCl(phen)_2_][Fe(CN)_5_NO]^.^DMF which consists of discrete [CuCl(phen)_2_]^+^ and [Fe(CN)_5_NO]^2-^ ions (Fig. 1) as well as dimethylformamide solvent molecules. The Cu^II^ ion adopts a distorted trigonal–bipyramidal environment by coordinating with four nitrogen atoms from two phen ligands and chlorine atom. The dihedral angle between the two phen ligands (77.92 (7)° ) as well as the range of Cu—N bond distances of 1.996 (3) - 2.177 (4) Å is in good agreement with the previously reported values for analagous complexes (Onawumi *et al.*, 2010; Sui *et al.*, 2006; Xiao *et al.*, 2004). The nitroprusside ion lies across an inversion center. Therefore, the CN and NO groups occupy the axial positions with equal occupancies. It has the usual distorted octahedral, pagoda-like, conformation with average Fe—C and Fe—N bond distances of 1.90 Å and 1.78 Å respectively, in a good agreement with literature values (Soria *et al.* (2002); Shevyakova *et al.* (2002). In the crystal, weak C—H···N and C—H···Cl hydrogen bonds connect anions and cations into a two-dimensional network parallel to (100) (Fig. 2). In addition, π···π stacking interactions are observed with centroid to centroid distances in the range 3.565 (2)–3.760 (3)Å.

### Experimental

Copper powder (0.159 g, 2.50 mmol), Na_2_[Fe(CN)_5_(NO)]^.^2H_2_O (0.372 g, 1.25 mmol) and phen^.^HCl^.^H_2_O (1.17 g, 5.00 mmol) in DMF (20 ml) were heated to 323–333 K and stirred magnetically until total dissolution of copper was observed (75 min). Green crystals suitable for X-ray crystallography were isolated in four days. The crystals (0.18 g, yield 14%) were filtered off, washed with dry methanol, and finally dried *in vacuo* at room temperature.

### Refinement

All H-atoms were placed in calculated positions with C—H = 0.93-1.00 Å and refined in riding-model approximation with U_iso_(H) = 1.2U_eq_(C).

### Crystal data

**Table:**

[CuCl(C_12_H_8_N_2_)_2_]_2_[Fe(CN)_5_(NO)]·C_3_H_7_NO	*V* = 1295.9 (4) Å^3^
*M**_r_* = 1207.85	*Z* = 1
Triclinic, *P*1	*F*(000) = 614
Hall symbol: -P 1	*D*_x_ = 1.548 Mg m^−^^3^
*a* = 9.9645 (13) Å	Mo *K*α radiation, λ = 0.71073 Å
*b* = 10.6001 (18) Å	µ = 1.25 mm^−^^1^
*c* = 12.623 (2) Å	*T* = 293 K
α = 79.585 (14)°	Block, green
β = 84.896 (12)°	0.50 × 0.40 × 0.20 mm
γ = 82.047 (12)°	

### Data collection

**Table:**

Oxford Diffraction Xcalibur 3 diffractometer	5647 independent reflections
Radiation source: fine-focus sealed tube	2685 reflections with *I* > 2σ(*I*)
Graphite monochromator	*R*_int_ = 0.041
Detector resolution: 16.1827 pixels mm^-1^	θ_max_ = 27.0°, θ_min_ = 3.9°
ω–scans	*h* = −12→12
Absorption correction: numerical (*CrysAlis PRO*; Oxford Diffraction, 2010)	*k* = −13→13
*T*_min_ = 0.575, *T*_max_ = 0.789	*l* = −16→16
11289 measured reflections	

### Refinement

**Table:**

Refinement on *F*^2^	Primary atom site location: structure-invariant direct methods
Least-squares matrix: full	Secondary atom site location: difference Fourier map
*R*[*F*^2^ > 2σ(*F*^2^)] = 0.061	Hydrogen site location: inferred from neighbouring sites
*wR*(*F*^2^) = 0.182	H-atom parameters constrained
*S* = 0.99	*w* = 1/[σ^2^(*F*_o_^2^) + (0.090*P*)^2^] where *P* = (*F*_o_^2^ + 2*F*_c_^2^)/3
5647 reflections	(Δ/σ)_max_ = 0.001
366 parameters	Δρ_max_ = 0.73 e Å^−^^3^
5 restraints	Δρ_min_ = −0.61 e Å^−^^3^

### Special details

**Table:**

Geometry. All e.s.d.'s (except the e.s.d. in the dihedral angle between two l.s. planes) are estimated using the full covariance matrix. The cell e.s.d.'s are taken into account individually in the estimation of e.s.d.'s in distances, angles and torsion angles; correlations between e.s.d.'s in cell parameters are only used when they are defined by crystal symmetry. An approximate (isotropic) treatment of cell e.s.d.'s is used for estimating e.s.d.'s involving l.s. planes.
Refinement. Refinement of *F*^2^ against ALL reflections. The weighted *R*-factor *wR* and goodness of fit *S* are based on *F*^2^, conventional *R*-factors *R* are based on *F*, with *F* set to zero for negative *F*^2^. The threshold expression of *F*^2^ > σ(*F*^2^) is used only for calculating *R*-factors(gt) *etc*. and is not relevant to the choice of reflections for refinement. *R*-factors based on *F*^2^ are statistically about twice as large as those based on *F*, and *R*- factors based on ALL data will be even larger.

### Fractional atomic coordinates and isotropic or equivalent isotropic displacement parameters (Å^2^)

**Table:**

	*x*	*y*	*z*	*U*_iso_*/*U*_eq_	Occ. (<1)
Cu1	0.17993 (5)	1.01179 (6)	0.25097 (5)	0.0663 (2)	
Fe1	0.5000	0.5000	0.5000	0.0698 (3)	
Cl1	0.04963 (14)	1.13793 (15)	0.12224 (12)	0.0907 (4)	
N1	0.3265 (4)	1.1266 (4)	0.2215 (3)	0.0620 (9)	
N2	0.3436 (4)	0.8814 (4)	0.1883 (3)	0.0672 (10)	
N3	0.0513 (3)	0.8798 (4)	0.2939 (3)	0.0644 (10)	
N4	0.2017 (3)	0.9736 (3)	0.4164 (3)	0.0567 (9)	
C25	0.6109 (5)	0.4836 (4)	0.3720 (5)	0.0657 (12)	
N5	0.6773 (4)	0.4748 (5)	0.2946 (4)	0.0842 (12)	
C26	0.3406 (5)	0.5273 (5)	0.4171 (4)	0.0672 (12)	
N6	0.2486 (4)	0.5418 (4)	0.3662 (4)	0.0821 (12)	
C27	0.514 (4)	0.696 (3)	0.474 (4)	0.066 (6)	0.50
N7	0.5204 (17)	0.796 (3)	0.446 (3)	0.107 (9)	0.50
N7B	0.507 (3)	0.660 (2)	0.471 (3)	0.057 (5)	0.50
O7B	0.517 (2)	0.776 (2)	0.457 (2)	0.123 (8)	0.50
C1	0.3150 (5)	1.2477 (5)	0.2380 (4)	0.0752 (13)	
H1	0.2321	1.2846	0.2654	0.090*	
C2	0.4228 (6)	1.3221 (6)	0.2157 (4)	0.0824 (15)	
H2	0.4134	1.4050	0.2323	0.099*	
C3	0.5410 (6)	1.2716 (6)	0.1697 (4)	0.0872 (17)	
H3	0.6121	1.3215	0.1520	0.105*	
C4	0.5573 (4)	1.1453 (6)	0.1485 (4)	0.0694 (13)	
C5	0.4460 (4)	1.0740 (5)	0.1772 (3)	0.0590 (11)	
C6	0.4538 (4)	0.9441 (5)	0.1594 (3)	0.0605 (11)	
C7	0.5754 (5)	0.8866 (6)	0.1118 (4)	0.0725 (14)	
C8	0.5751 (7)	0.7591 (7)	0.0950 (4)	0.0942 (19)	
H8	0.6514	0.7168	0.0625	0.113*	
C9	0.4641 (7)	0.6979 (6)	0.1260 (5)	0.0972 (18)	
H9	0.4648	0.6129	0.1163	0.117*	
C10	0.3494 (6)	0.7617 (5)	0.1723 (4)	0.0829 (15)	
H10	0.2737	0.7183	0.1928	0.100*	
C11	0.6773 (5)	1.0833 (8)	0.1005 (4)	0.0872 (17)	
H11	0.7523	1.1281	0.0820	0.105*	
C12	0.6851 (5)	0.9618 (7)	0.0812 (4)	0.0874 (18)	
H12	0.7640	0.9259	0.0471	0.105*	
C13	−0.0238 (5)	0.8380 (5)	0.2298 (5)	0.0808 (15)	
H13	−0.0229	0.8740	0.1569	0.097*	
C14	−0.1058 (5)	0.7390 (5)	0.2707 (5)	0.0840 (15)	
H14	−0.1585	0.7106	0.2246	0.101*	
C15	−0.1078 (5)	0.6856 (5)	0.3758 (5)	0.0766 (14)	
H15	−0.1617	0.6204	0.4027	0.092*	
C16	−0.0292 (4)	0.7284 (4)	0.4436 (4)	0.0641 (12)	
C17	0.0489 (4)	0.8265 (4)	0.3995 (4)	0.0600 (11)	
C18	0.1300 (4)	0.8767 (4)	0.4657 (4)	0.0559 (10)	
C19	0.1308 (4)	0.8260 (4)	0.5758 (4)	0.0611 (11)	
C20	0.2095 (5)	0.8823 (5)	0.6371 (4)	0.0741 (13)	
H20	0.2116	0.8539	0.7111	0.089*	
C21	0.2825 (5)	0.9787 (5)	0.5879 (4)	0.0753 (13)	
H21	0.3356	1.0154	0.6281	0.090*	
C22	0.2776 (4)	1.0221 (5)	0.4772 (4)	0.0655 (12)	
H22	0.3290	1.0872	0.4446	0.079*	
C23	−0.0211 (5)	0.6775 (5)	0.5573 (4)	0.0723 (13)	
H23	−0.0690	0.6091	0.5880	0.087*	
C24	0.0522 (5)	0.7251 (5)	0.6187 (4)	0.0745 (14)	
H24	0.0523	0.6916	0.6920	0.089*	
N8	0.0000	0.5000	1.0000	0.157 (4)*	
O1	−0.2091 (11)	0.465 (2)	1.004 (2)	0.273 (9)*	0.50
C28	−0.1053 (13)	0.483 (3)	0.9484 (13)	0.213 (10)*	0.50
H28A	−0.1030	0.4841	0.8688	0.256*	0.50
C29	0.015 (2)	0.504 (2)	1.1107 (9)	0.177 (8)*	0.50
H29A	−0.0724	0.4960	1.1561	0.212*	0.50
H29B	0.0843	0.4305	1.1378	0.212*	0.50
H29C	0.0475	0.5876	1.1150	0.212*	0.50
C30	0.1174 (18)	0.512 (2)	0.9282 (17)	0.186 (8)*	0.50
H30A	0.0824	0.5077	0.8573	0.223*	0.50
H30B	0.1544	0.5962	0.9235	0.223*	0.50
H30C	0.1912	0.4391	0.9462	0.223*	0.50

### Atomic displacement parameters (Å^2^)

**Table:**

	*U*^11^	*U*^22^	*U*^33^	*U*^12^	*U*^13^	*U*^23^
Cu1	0.0579 (3)	0.0790 (4)	0.0627 (4)	−0.0218 (3)	0.0013 (3)	−0.0064 (3)
Fe1	0.0523 (5)	0.0874 (7)	0.0730 (7)	−0.0208 (5)	0.0005 (4)	−0.0155 (5)
Cl1	0.0809 (8)	0.1060 (11)	0.0834 (9)	−0.0154 (7)	−0.0016 (7)	−0.0103 (8)
N1	0.058 (2)	0.071 (3)	0.059 (2)	−0.0179 (18)	0.0037 (17)	−0.010 (2)
N2	0.066 (2)	0.077 (3)	0.053 (2)	−0.014 (2)	−0.0005 (18)	0.003 (2)
N3	0.057 (2)	0.072 (2)	0.065 (2)	−0.0181 (18)	−0.0070 (18)	−0.005 (2)
N4	0.0478 (17)	0.064 (2)	0.059 (2)	−0.0160 (16)	0.0040 (16)	−0.0101 (18)
C25	0.053 (2)	0.063 (3)	0.084 (3)	−0.010 (2)	−0.010 (2)	−0.016 (3)
N5	0.065 (2)	0.105 (3)	0.084 (3)	−0.012 (2)	0.001 (2)	−0.025 (3)
C26	0.057 (3)	0.063 (3)	0.077 (3)	−0.015 (2)	0.013 (2)	−0.003 (3)
N6	0.062 (2)	0.096 (3)	0.085 (3)	−0.021 (2)	−0.003 (2)	0.001 (3)
C27	0.072 (9)	0.048 (17)	0.091 (11)	−0.030 (11)	0.006 (7)	−0.033 (14)
N7	0.060 (7)	0.068 (14)	0.19 (2)	−0.003 (7)	−0.031 (10)	0.001 (11)
N7B	0.047 (5)	0.043 (12)	0.092 (8)	−0.016 (8)	−0.010 (5)	−0.028 (10)
O7B	0.186 (15)	0.047 (8)	0.140 (13)	−0.058 (8)	0.040 (10)	−0.017 (9)
C1	0.073 (3)	0.082 (4)	0.072 (3)	−0.020 (3)	0.009 (3)	−0.014 (3)
C2	0.094 (4)	0.086 (4)	0.073 (3)	−0.035 (3)	0.009 (3)	−0.019 (3)
C3	0.089 (4)	0.111 (5)	0.072 (3)	−0.058 (3)	−0.008 (3)	−0.009 (3)
C4	0.054 (2)	0.106 (4)	0.050 (3)	−0.021 (3)	−0.006 (2)	−0.005 (3)
C5	0.050 (2)	0.084 (3)	0.043 (2)	−0.021 (2)	−0.0032 (18)	−0.001 (2)
C6	0.063 (3)	0.076 (3)	0.040 (2)	−0.004 (2)	−0.009 (2)	−0.004 (2)
C7	0.065 (3)	0.096 (4)	0.051 (3)	0.006 (3)	−0.016 (2)	−0.003 (3)
C8	0.098 (4)	0.106 (5)	0.065 (3)	0.032 (4)	−0.010 (3)	−0.010 (3)
C9	0.132 (5)	0.075 (4)	0.075 (4)	0.007 (4)	−0.009 (4)	0.001 (3)
C10	0.109 (4)	0.071 (4)	0.066 (3)	−0.020 (3)	−0.003 (3)	−0.001 (3)
C11	0.053 (3)	0.152 (6)	0.059 (3)	−0.026 (3)	0.000 (2)	−0.014 (4)
C12	0.052 (3)	0.152 (6)	0.056 (3)	0.003 (3)	−0.008 (2)	−0.019 (4)
C13	0.070 (3)	0.100 (4)	0.079 (3)	−0.023 (3)	−0.015 (3)	−0.015 (3)
C14	0.075 (3)	0.091 (4)	0.096 (4)	−0.033 (3)	−0.015 (3)	−0.023 (3)
C15	0.056 (3)	0.071 (3)	0.104 (4)	−0.014 (2)	−0.007 (3)	−0.012 (3)
C16	0.045 (2)	0.059 (3)	0.087 (3)	−0.011 (2)	0.003 (2)	−0.010 (3)
C17	0.047 (2)	0.062 (3)	0.069 (3)	−0.0097 (19)	0.000 (2)	−0.007 (2)
C18	0.044 (2)	0.062 (3)	0.061 (3)	−0.0095 (19)	0.0022 (19)	−0.008 (2)
C19	0.052 (2)	0.067 (3)	0.061 (3)	−0.007 (2)	0.003 (2)	−0.004 (2)
C20	0.074 (3)	0.092 (4)	0.055 (3)	−0.013 (3)	−0.002 (2)	−0.010 (3)
C21	0.076 (3)	0.089 (4)	0.068 (3)	−0.021 (3)	−0.011 (3)	−0.020 (3)
C22	0.062 (2)	0.071 (3)	0.065 (3)	−0.018 (2)	−0.001 (2)	−0.007 (2)
C23	0.063 (3)	0.070 (3)	0.079 (3)	−0.020 (2)	0.009 (3)	0.003 (3)
C24	0.067 (3)	0.079 (3)	0.070 (3)	−0.014 (3)	0.009 (2)	0.004 (3)

### Geometric parameters (Å, º)

**Table:**

Cu1—N1	1.996 (3)	C11—H11	0.9300
Cu1—N3	1.998 (4)	C12—H12	0.9300
Cu1—N4	2.079 (4)	C13—C14	1.415 (7)
Cu1—N2	2.177 (4)	C13—H13	0.9300
Cu1—Cl1	2.2855 (16)	C14—C15	1.345 (7)
Fe1—N7B	1.68 (2)	C14—H14	0.9300
Fe1—N7B^i^	1.68 (2)	C15—C16	1.382 (7)
Fe1—C25^i^	1.895 (6)	C15—H15	0.9300
Fe1—C25	1.895 (6)	C16—C17	1.391 (6)
Fe1—C26^i^	1.939 (6)	C16—C23	1.444 (7)
Fe1—C26	1.939 (6)	C17—C18	1.427 (6)
Fe1—C27	2.07 (3)	C18—C19	1.397 (6)
Fe1—C27^i^	2.07 (3)	C19—C20	1.405 (7)
N1—C1	1.325 (6)	C19—C24	1.413 (6)
N1—C5	1.361 (5)	C20—C21	1.362 (7)
N2—C10	1.313 (6)	C20—H20	0.9300
N2—C6	1.351 (6)	C21—C22	1.393 (7)
N3—C13	1.320 (6)	C21—H21	0.9300
N3—C17	1.350 (6)	C22—H22	0.9300
N4—C22	1.334 (6)	C23—C24	1.317 (7)
N4—C18	1.358 (5)	C23—H23	0.9300
C25—N5	1.141 (6)	C24—H24	0.9300
C26—N6	1.143 (6)	N8—C28^ii^	1.333 (3)
C27—O7B	0.83 (4)	N8—C28	1.333 (3)
C27—N7	1.06 (4)	N8—C30^ii^	1.421 (9)
N7—N7B	1.45 (4)	N8—C30	1.421 (9)
N7B—O7B	1.23 (3)	N8—C29	1.426 (9)
C1—C2	1.398 (7)	N8—C29^ii^	1.426 (9)
C1—H1	0.9300	O1—C28	1.212 (3)
C2—C3	1.351 (8)	O1—C30^ii^	1.38 (3)
C2—H2	0.9300	C28—C29^ii^	1.13 (2)
C3—C4	1.397 (8)	C28—C30^ii^	1.56 (2)
C3—H3	0.9300	C28—H28A	1.0001
C4—C5	1.412 (6)	C29—C28^ii^	1.13 (2)
C4—C11	1.423 (8)	C29—C30^ii^	1.49 (2)
C5—C6	1.425 (7)	C29—H29A	1.0000
C6—C7	1.414 (6)	C29—H29B	0.9999
C7—C8	1.406 (8)	C29—H29C	1.0000
C7—C12	1.426 (8)	C30—O1^ii^	1.38 (3)
C8—C9	1.352 (8)	C30—C29^ii^	1.49 (2)
C8—H8	0.9300	C30—C28^ii^	1.56 (2)
C9—C10	1.383 (8)	C30—H30A	1.0000
C9—H9	0.9300	C30—H30B	1.0000
C10—H10	0.9300	C30—H30C	1.0000
C11—C12	1.344 (8)		
			
N1—Cu1—N3	171.91 (15)	N3—C13—H13	119.6
N1—Cu1—N4	93.42 (14)	C14—C13—H13	119.6
N3—Cu1—N4	80.94 (14)	C15—C14—C13	120.3 (5)
N1—Cu1—N2	80.03 (16)	C15—C14—H14	119.8
N3—Cu1—N2	95.56 (15)	C13—C14—H14	119.8
N4—Cu1—N2	103.41 (14)	C14—C15—C16	119.4 (5)
N1—Cu1—Cl1	92.87 (12)	C14—C15—H15	120.3
N3—Cu1—Cl1	95.13 (11)	C16—C15—H15	120.3
N4—Cu1—Cl1	142.15 (10)	C15—C16—C17	118.0 (5)
N2—Cu1—Cl1	114.45 (11)	C15—C16—C23	124.6 (4)
N7B—Fe1—N7B^i^	180.000 (3)	C17—C16—C23	117.4 (4)
N7B—Fe1—C25^i^	92.0 (13)	N3—C17—C16	122.6 (4)
N7B^i^—Fe1—C25^i^	88.0 (13)	N3—C17—C18	116.7 (4)
N7B—Fe1—C25	88.0 (13)	C16—C17—C18	120.6 (4)
N7B^i^—Fe1—C25	92.0 (13)	N4—C18—C19	123.8 (4)
C25^i^—Fe1—C25	180.000 (1)	N4—C18—C17	116.8 (4)
N7B—Fe1—C26^i^	91.7 (10)	C19—C18—C17	119.3 (4)
N7B^i^—Fe1—C26^i^	88.3 (10)	C18—C19—C20	116.5 (4)
C25^i^—Fe1—C26^i^	89.34 (19)	C18—C19—C24	119.1 (4)
C25—Fe1—C26^i^	90.66 (19)	C20—C19—C24	124.4 (5)
N7B—Fe1—C26	88.3 (10)	C21—C20—C19	119.9 (5)
N7B^i^—Fe1—C26	91.7 (10)	C21—C20—H20	120.1
C25^i^—Fe1—C26	90.66 (19)	C19—C20—H20	120.1
C25—Fe1—C26	89.34 (19)	C20—C21—C22	119.8 (5)
C26^i^—Fe1—C26	180.000 (1)	C20—C21—H21	120.1
N7B—Fe1—C27	4 (2)	C22—C21—H21	120.1
N7B^i^—Fe1—C27	176 (2)	N4—C22—C21	122.2 (4)
C25^i^—Fe1—C27	89.7 (13)	N4—C22—H22	118.9
C25—Fe1—C27	90.3 (13)	C21—C22—H22	118.9
C26^i^—Fe1—C27	88.5 (12)	C24—C23—C16	121.9 (4)
C26—Fe1—C27	91.5 (12)	C24—C23—H23	119.1
N7B—Fe1—C27^i^	176 (2)	C16—C23—H23	119.1
N7B^i^—Fe1—C27^i^	4 (2)	C23—C24—C19	121.6 (5)
C25^i^—Fe1—C27^i^	90.3 (13)	C23—C24—H24	119.2
C25—Fe1—C27^i^	89.7 (13)	C19—C24—H24	119.2
C26^i^—Fe1—C27^i^	91.5 (12)	C28^ii^—N8—C28	180.000 (4)
C26—Fe1—C27^i^	88.5 (12)	C28^ii^—N8—C30^ii^	111.0 (11)
C27—Fe1—C27^i^	180.000 (4)	C28—N8—C30^ii^	69.0 (11)
C1—N1—C5	118.9 (4)	C28^ii^—N8—C30	69.0 (11)
C1—N1—Cu1	125.8 (3)	C28—N8—C30	111.0 (11)
C5—N1—Cu1	115.3 (3)	C30^ii^—N8—C30	180.000 (5)
C10—N2—C6	118.9 (4)	C28^ii^—N8—C29	48.1 (10)
C10—N2—Cu1	131.9 (4)	C28—N8—C29	131.9 (10)
C6—N2—Cu1	109.2 (3)	C30^ii^—N8—C29	62.9 (11)
C13—N3—C17	118.9 (4)	C30—N8—C29	117.1 (11)
C13—N3—Cu1	126.9 (4)	C28^ii^—N8—C29^ii^	131.9 (10)
C17—N3—Cu1	114.1 (3)	C28—N8—C29^ii^	48.1 (10)
C22—N4—C18	117.7 (4)	C30^ii^—N8—C29^ii^	117.1 (11)
C22—N4—Cu1	131.1 (3)	C30—N8—C29^ii^	62.9 (11)
C18—N4—Cu1	111.1 (3)	C29—N8—C29^ii^	180.000 (7)
N5—C25—Fe1	179.4 (5)	C28—O1—C30^ii^	73.7 (8)
N6—C26—Fe1	178.2 (5)	C29^ii^—C28—O1	173.4 (12)
O7B—C27—N7	4 (3)	C29^ii^—C28—N8	70.3 (7)
O7B—C27—Fe1	174 (5)	O1—C28—N8	116.3 (9)
N7—C27—Fe1	170 (4)	C29^ii^—C28—C30^ii^	128.3 (8)
C27—N7—N7B	7 (4)	O1—C28—C30^ii^	58.2 (9)
O7B—N7B—N7	4 (2)	N8—C28—C30^ii^	58.1 (7)
O7B—N7B—Fe1	175 (3)	C29^ii^—C28—H28A	53.9
N7—N7B—Fe1	177 (2)	O1—C28—H28A	119.6
C27—O7B—N7B	7 (5)	N8—C28—H28A	124.1
N1—C1—C2	122.5 (5)	C30^ii^—C28—H28A	175.9
N1—C1—H1	118.7	C28^ii^—C29—N8	61.6 (6)
C2—C1—H1	118.7	C28^ii^—C29—C30^ii^	120.0 (10)
C3—C2—C1	118.9 (5)	N8—C29—C30^ii^	58.4 (7)
C3—C2—H2	120.5	C28^ii^—C29—H29A	173.5
C1—C2—H2	120.5	N8—C29—H29A	112.6
C2—C3—C4	120.7 (5)	C30^ii^—C29—H29A	54.2
C2—C3—H3	119.7	C28^ii^—C29—H29B	76.0
C4—C3—H3	119.7	N8—C29—H29B	107.6
C3—C4—C5	117.3 (4)	C30^ii^—C29—H29B	124.3
C3—C4—C11	125.0 (5)	H29A—C29—H29B	109.5
C5—C4—C11	117.7 (5)	C28^ii^—C29—H29C	71.1
N1—C5—C4	121.6 (4)	N8—C29—H29C	108.2
N1—C5—C6	117.2 (4)	C30^ii^—C29—H29C	126.2
C4—C5—C6	121.2 (4)	H29A—C29—H29C	109.5
N2—C6—C7	122.8 (5)	H29B—C29—H29C	109.5
N2—C6—C5	118.2 (4)	O1^ii^—C30—N8	100.9 (13)
C7—C6—C5	119.0 (4)	O1^ii^—C30—C29^ii^	159.1 (13)
C8—C7—C6	115.9 (5)	N8—C30—C29^ii^	58.7 (7)
C8—C7—C12	125.3 (5)	O1^ii^—C30—C28^ii^	48.1 (8)
C6—C7—C12	118.7 (5)	N8—C30—C28^ii^	52.8 (6)
C9—C8—C7	120.1 (5)	C29^ii^—C30—C28^ii^	111.5 (8)
C9—C8—H8	119.9	O1^ii^—C30—H30A	156.1
C7—C8—H8	119.9	N8—C30—H30A	102.4
C8—C9—C10	120.1 (6)	C29^ii^—C30—H30A	43.6
C8—C9—H9	120.0	C28^ii^—C30—H30A	155.1
C10—C9—H9	120.0	O1^ii^—C30—H30B	55.3
N2—C10—C9	122.2 (5)	N8—C30—H30B	113.5
N2—C10—H10	118.9	C29^ii^—C30—H30B	125.5
C9—C10—H10	118.9	C28^ii^—C30—H30B	82.6
C12—C11—C4	121.8 (5)	H30A—C30—H30B	109.5
C12—C11—H11	119.1	O1^ii^—C30—H30C	65.8
C4—C11—H11	119.1	N8—C30—H30C	112.3
C11—C12—C7	121.6 (5)	C29^ii^—C30—H30C	123.5
C11—C12—H12	119.2	C28^ii^—C30—H30C	85.5
C7—C12—H12	119.2	H30A—C30—H30C	109.5
N3—C13—C14	120.7 (5)	H30B—C30—H30C	109.5
			
N4—Cu1—N1—C1	−77.5 (4)	C17—N3—C13—C14	0.0 (7)
N2—Cu1—N1—C1	179.5 (4)	Cu1—N3—C13—C14	176.8 (4)
Cl1—Cu1—N1—C1	65.2 (4)	N3—C13—C14—C15	−0.2 (8)
N4—Cu1—N1—C5	104.9 (3)	C13—C14—C15—C16	0.1 (8)
N2—Cu1—N1—C5	1.8 (3)	C14—C15—C16—C17	0.3 (7)
Cl1—Cu1—N1—C5	−112.5 (3)	C14—C15—C16—C23	−178.9 (5)
N1—Cu1—N2—C10	−179.7 (5)	C13—N3—C17—C16	0.4 (7)
N3—Cu1—N2—C10	7.2 (5)	Cu1—N3—C17—C16	−176.8 (3)
N4—Cu1—N2—C10	89.2 (5)	C13—N3—C17—C18	−178.9 (4)
Cl1—Cu1—N2—C10	−91.0 (5)	Cu1—N3—C17—C18	3.9 (5)
N1—Cu1—N2—C6	−2.1 (3)	C15—C16—C17—N3	−0.5 (7)
N3—Cu1—N2—C6	−175.2 (3)	C23—C16—C17—N3	178.8 (4)
N4—Cu1—N2—C6	−93.2 (3)	C15—C16—C17—C18	178.7 (4)
Cl1—Cu1—N2—C6	86.6 (3)	C23—C16—C17—C18	−2.0 (6)
N4—Cu1—N3—C13	178.4 (4)	C22—N4—C18—C19	0.4 (6)
N2—Cu1—N3—C13	−78.8 (4)	Cu1—N4—C18—C19	176.7 (3)
Cl1—Cu1—N3—C13	36.4 (4)	C22—N4—C18—C17	179.7 (4)
N4—Cu1—N3—C17	−4.6 (3)	Cu1—N4—C18—C17	−3.9 (4)
N2—Cu1—N3—C17	98.1 (3)	N3—C17—C18—N4	0.2 (6)
Cl1—Cu1—N3—C17	−146.6 (3)	C16—C17—C18—N4	−179.1 (4)
N1—Cu1—N4—C22	6.1 (4)	N3—C17—C18—C19	179.6 (4)
N3—Cu1—N4—C22	−179.7 (4)	C16—C17—C18—C19	0.3 (6)
N2—Cu1—N4—C22	86.7 (4)	N4—C18—C19—C20	1.2 (6)
Cl1—Cu1—N4—C22	−93.0 (4)	C17—C18—C19—C20	−178.1 (4)
N1—Cu1—N4—C18	−169.6 (3)	N4—C18—C19—C24	180.0 (4)
N3—Cu1—N4—C18	4.6 (3)	C17—C18—C19—C24	0.7 (6)
N2—Cu1—N4—C18	−89.0 (3)	C18—C19—C20—C21	−1.8 (7)
Cl1—Cu1—N4—C18	91.3 (3)	C24—C19—C20—C21	179.5 (4)
C5—N1—C1—C2	−2.7 (7)	C19—C20—C21—C22	0.8 (8)
Cu1—N1—C1—C2	179.7 (4)	C18—N4—C22—C21	−1.5 (7)
N1—C1—C2—C3	4.0 (8)	Cu1—N4—C22—C21	−176.9 (3)
C1—C2—C3—C4	−2.5 (8)	C20—C21—C22—N4	0.9 (8)
C2—C3—C4—C5	0.0 (8)	C15—C16—C23—C24	−177.7 (5)
C2—C3—C4—C11	−180.0 (5)	C17—C16—C23—C24	3.0 (7)
C1—N1—C5—C4	0.0 (6)	C16—C23—C24—C19	−2.2 (8)
Cu1—N1—C5—C4	177.8 (3)	C18—C19—C24—C23	0.3 (7)
C1—N1—C5—C6	−179.2 (4)	C20—C19—C24—C23	179.0 (5)
Cu1—N1—C5—C6	−1.4 (5)	C30^ii^—O1—C28—N8	3 (2)
C3—C4—C5—N1	1.4 (6)	C30^ii^—N8—C28—C29^ii^	178 (3)
C11—C4—C5—N1	−178.7 (4)	C30—N8—C28—C29^ii^	−2 (3)
C3—C4—C5—C6	−179.5 (4)	C29—N8—C28—C29^ii^	180.000 (3)
C11—C4—C5—C6	0.4 (6)	C28^ii^—N8—C28—O1	171 (100)
C10—N2—C6—C7	0.5 (7)	C30^ii^—N8—C28—O1	−3 (2)
Cu1—N2—C6—C7	−177.5 (3)	C30—N8—C28—O1	177 (2)
C10—N2—C6—C5	180.0 (4)	C29—N8—C28—O1	−1 (4)
Cu1—N2—C6—C5	2.0 (5)	C29^ii^—N8—C28—O1	179 (4)
N1—C5—C6—N2	−0.6 (6)	C30—N8—C28—C30^ii^	180.000 (5)
C4—C5—C6—N2	−179.8 (4)	C29—N8—C28—C30^ii^	2 (3)
N1—C5—C6—C7	178.9 (4)	C29^ii^—N8—C28—C30^ii^	−178 (3)
C4—C5—C6—C7	−0.2 (6)	C28—N8—C29—C28^ii^	180.000 (4)
N2—C6—C7—C8	0.5 (7)	C30^ii^—N8—C29—C28^ii^	−177 (3)
C5—C6—C7—C8	−179.0 (4)	C30—N8—C29—C28^ii^	3 (3)
N2—C6—C7—C12	178.2 (4)	C28^ii^—N8—C29—C30^ii^	177 (3)
C5—C6—C7—C12	−1.3 (6)	C28—N8—C29—C30^ii^	−3 (3)
C6—C7—C8—C9	−1.5 (8)	C30—N8—C29—C30^ii^	180.000 (4)
C12—C7—C8—C9	−179.0 (5)	C28^ii^—N8—C30—O1^ii^	−2.8 (19)
C7—C8—C9—C10	1.4 (9)	C28—N8—C30—O1^ii^	177.2 (19)
C6—N2—C10—C9	−0.6 (8)	C29—N8—C30—O1^ii^	−5 (2)
Cu1—N2—C10—C9	176.9 (4)	C29^ii^—N8—C30—O1^ii^	175 (2)
C8—C9—C10—N2	−0.4 (9)	C28^ii^—N8—C30—C29^ii^	−178 (2)
C3—C4—C11—C12	−179.1 (5)	C28—N8—C30—C29^ii^	2 (2)
C5—C4—C11—C12	1.0 (7)	C29—N8—C30—C29^ii^	180.000 (4)
C4—C11—C12—C7	−2.6 (8)	C28—N8—C30—C28^ii^	180.000 (4)
C8—C7—C12—C11	−179.8 (5)	C29—N8—C30—C28^ii^	−2 (2)
C6—C7—C12—C11	2.7 (7)	C29^ii^—N8—C30—C28^ii^	178 (2)

Symmetry codes: (i) −*x*+1, −*y*+1, −*z*+1; (ii) −*x*, −*y*+1, −*z*+2.

### Hydrogen-bond geometry (Å, º)

**Table:**

*D*—H···*A*	*D*—H	H···*A*	*D*···*A*	*D*—H···*A*
C12—H12···Cl1^iii^	0.93	2.82	3.701 (6)	159
C23—H23···N6^iv^	0.93	2.52	3.425 (7)	163

Symmetry codes: (iii) −*x*+1, −*y*+2, −*z*; (iv) −*x*, −*y*+1, −*z*+1.
